# Immune Function and Diversity of Osteoclasts in Normal and Pathological Conditions

**DOI:** 10.3389/fimmu.2019.01408

**Published:** 2019-06-19

**Authors:** Maria-Bernadette Madel, Lidia Ibáñez, Abdelilah Wakkach, Teun J. de Vries, Anna Teti, Florence Apparailly, Claudine Blin-Wakkach

**Affiliations:** ^1^CNRS, Laboratoire de PhysioMédecine Moléculaire, Faculté de Médecine, UMR7370, Nice, France; ^2^Faculé de Médecine, Université Côte d'Azur, Nice, France; ^3^Department of Pharmacy, Cardenal Herrera-CEU University, València, Spain; ^4^Department of Periodontology, Academic Centre of Dentistry Amsterdam, University of Amsterdam and Vrije Univeristeit, Amsterdam, Netherlands; ^5^Department of Biotechnological and Applied Clinical Sciences, University of L'Aquila, L'Aquila, Italy; ^6^IRMB, INSERM, CHU Montpellier, Université Montpellier, Montpellier, France

**Keywords:** osteoclast, osteoimmunology, monocyte heterogeneity, inflammation, immune modulation, dendritic cell

## Abstract

Osteoclasts (OCLs) are key players in controlling bone remodeling. Modifications in their differentiation or bone resorbing activity are associated with a number of pathologies ranging from osteopetrosis to osteoporosis, chronic inflammation and cancer, that are all characterized by immunological alterations. Therefore, the 2000s were marked by the emergence of osteoimmunology and by a growing number of studies focused on the control of OCL differentiation and function by the immune system. At the same time, it was discovered that OCLs are much more than bone resorbing cells. As monocytic lineage-derived cells, they belong to a family of cells that displays a wide heterogeneity and plasticity and that is involved in phagocytosis and innate immune responses. However, while OCLs have been extensively studied for their bone resorption capacity, their implication as immune cells was neglected for a long time. In recent years, new evidence pointed out that OCLs play important roles in the modulation of immune responses toward immune suppression or inflammation. They unlocked their capacity to modulate T cell activation, to efficiently process and present antigens as well as their ability to activate T cell responses in an antigen-dependent manner. Moreover, similar to other monocytic lineage cells such as macrophages, monocytes and dendritic cells, OCLs display a phenotypic and functional plasticity participating to their anti-inflammatory or pro-inflammatory effect depending on their cell origin and environment. This review will address this novel vision of the OCL, not only as a phagocyte specialized in bone resorption, but also as innate immune cell participating in the control of immune responses.

## Introduction

Bone-resorbing osteoclasts (OCLs) were first described 150 years ago (1). Their origin remained unclear for nearly 100 years before they were formally identified as cells of hematopoietic origin. This was evidenced thanks to the analysis of osteopetrotic mice defective in osteoclast function. Hematopoietic cell transfer from normal littermates restored OCL function in *mi/mi* mice and reciprocal transfer of hematopoietic cells from *mi/mi* mice induced osteopetrosis in normal recipient mice (2). The monocytic origin of OCLs was first demonstrated in colony assays of bone marrow cell fractions (3). From this moment, OCLs have been extensively studied to decipher the mechanisms of bone resorption leading to the identification of key factors required for OCL differentiation, fusion, bone adhesion and bone degradation activity. These studies defined a set of specific properties that cells must fulfill to be defined as *bona fide* OCLs, the most important being multinucleation, the expression of markers such as the tartrate-resistant acid phosphatase (TRAcP) and the capacity to degrade bone and mineralized matrix (4).

Among hematopoietic cells, OCLs belong to the monocytic family. This family of innate immune cells is characterized by its capacity to sense and respond to infections and tissue damage, its phagocytic properties and its high plasticity controlled by the tissue micro-environmental heterogeneity (5–7). Abundant literature addressed the origins and roles of monocytes (MNs), macrophages (Mϕs), and dendritic cells (DCs). Nowadays, it is clearly established that each of these populations includes distinct sub-groups that have specific origin and functional properties ranging from inflammatory to immune suppressive effects (8, 9). However, despite their common origin, the potential implication of OCLs as innate immune cells has been neglected for a long time. The immune face of OCLs emerged only 10 years ago when costimulatory signals mediated by ITAM motifs involved in immune cell activation were shown to be essential for OCL differentiation (10–12). This was further emphasized by the identification of the important link between DCs and OCLs through the ability of DCs to differentiate into bone-resorbing OCLs under pathological conditions (13, 14) (Table 1).

**Table 1:** Pathological conditions associated with inflammatory osteoclasts differentiated from dendritic cells.

**DC origin**	**Pathological condition**	**Demonstrated**	**References**
Murine splenic cDCs	Osteopetrosis, high Th17 proportion	*In vivo, in vitro*	(14, 15)
Murine BM-derived DCs	Calvaria induced osteolysis	*In vivo*	(16)
Murine splenic DCs (cell line)	Histiocytosis	*In vivo, in vitro*	(17)
Murine BM-derived DCs	Chronic inflammation	*In vitro*	(18)
Murine Flt3-L-induced BM-derived cDCs	+IL-1β and TNF-α	*In vitro*	(19)
Murine BM-derived DCs and splenic DCs	Periodontitis	*In vitro*	(20)
Human blood-derived MNs	Rheumatoid arthritis	*In vitro*	(13)
Human BM DCs	Multiple myeloma, high level of IL-17	*In vitro*	(21, 22)
Human Langerhans cells^*^	Langerhans cell histiocytosis	*In vivo*	(23)

* *Although Langerhans cells function as DCs, they are related to macrophages in terms of their origin (24–26)*.

**Figure 1:**
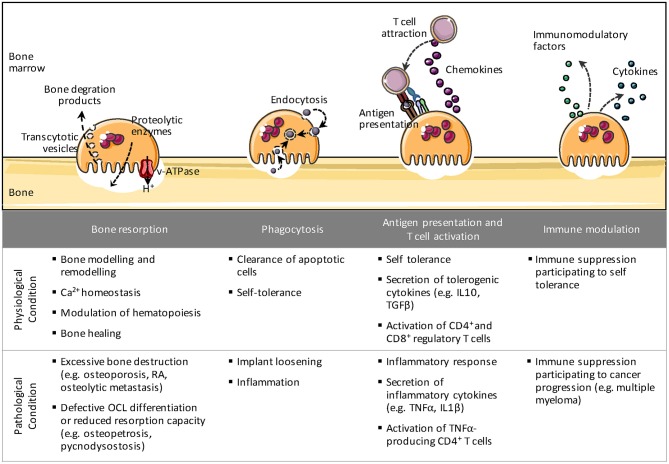
The different immune-related roles of osteoclasts. Besides bone resorption, osteoclasts share many properties with their precursor cells such as phagocytosis, antigen presentation, and immune modulation. These immune-related functions may possess divergent functionalities under physiological and pathological conditions.

In fact, OCLs share many similarities with Mϕs and DCs in their origin and function (Figure 1). Like Mϕs in tissues, OCLs are essential to maintain bone homeostasis and remodeling in steady state and to support bone healing after bone damage. Beside bone matrix resorption, they are able to take up apoptotic cells, calcium-phosphate particles or latex beads (27–31). More recently, OCLs have been shown to process, present and cross-present antigens resulting in T cell activation (18, 32, 33). They also produce cytokines and immunomodulatory factors that affect immune responses (34–36), as described below. Moreover, like their monocytic counterparts, OCLs display phenotypic heterogeneity and may arise from different progenitors depending on their environment, the stimuli they receive and their developmental stage (18, 37, 38). In particular, pathological conditions associated to inflammation or cancer provide molecular and cellular signals that stimulate specific monocytic subsets to differentiate into OCLs. Despite this heterogeneity in their origin and environment, OCLs are largely considered as a single population of cells. Consequently, thus far, under pathological conditions OCLs have been investigated for their increased or decreased differentiation and resorptive function but almost never with regard to the implication of different OCL subsets. Moreover, the immunological function of OCLs remains poorly explored. This review addresses the state-of-the-art of this novel vision of the OCL not just as a bone-resorbing cell but also as a cell having immune capacities.

## Diversity in Osteoclast Origin

### Bone Marrow Osteoclast Progenitors

**Figure 2:**
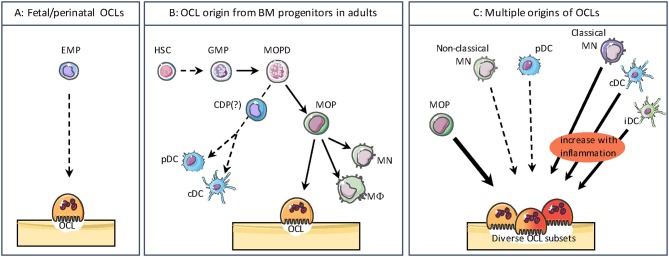
The different origins of osteoclasts. **(A)** During the embryonic and postnatal period, OCLs differentiate from the embryonic erythro-myeloid progenitor (EMP) lineage. **(B)** In adults, bone marrow progenitors for osteoclasts (OCLs) and other monocytic cells such as monocytes (MN), macrophages (MΦ), as well as conventional/classical dendritic cells (cDCs) and plasmacytoid DCs (pDCs) share common progenitors downstream of the granulocyte/MΦ progenitor (GMP). HSC, Hematopoietic Stem Cell; MOPD, MΦ/OCL/DC progenitor; CDP, common DC progenitor; MOP, MΦ/OC progenitor. The divergence of CDP from MODP is not clearly evidenced. **(C)** OCLs have multiple origins depending on their environment. Beside their origin from MOP, in pathological conditions OCLs can also arise from classical MNs, cDCs and inflammatory DCs (iDC). OCLs differentiation from non-classical and pDCs is much less efficient. These different sources of OCLs result in a heterogeneity as observed for other monocytic cells.

Whereas, MN, Mϕ, and DC origin has been widely explored, the origin of OCLs remained more elusive and OCLs are usually forgotten in hematopoietic lineage trees. However, in adults a common early bone marrow (BM) progenitor for OCLs and other monocytic cells (Mϕ/OCL/DC progenitor, MODP) has been identified downstream of the granulocyte/Mϕ progenitor (GMP) (Figure 2) (39, 40). In human, CD11b^−^CD34^+^c-KIT^+^FLT3^+^IL-3Rα^low^ BM cells are common progenitors for Mϕs, DCs, OCLs and granulocytes. They give rise to CD11b^−^CD34^+^c-KIT^+^FLT3^+^IL-3Rα^high^ cells that are restricted to Mϕ, DC, and OCL differentiation and represent <0.5% of BM cells (39). In mouse, the BM CD11b^−/low^c-kit^+^CD115^+^ fraction contains a common precursor for Mϕs, OCLs, and DCs also representing <0.5% of BM cells (41–44). Within this population, the use of CD27 and Flt3 markers further discriminates subsets of oligopotent progenitors for Mϕ/OCL/DC development (CD27^+^Flt3^+^) vs. bipotent progenitors for Mϕ/OCL development (CD27^low/−^Flt3^−^) (40, 45). Moreover, analysis of mouse BM myeloid fractions revealed that early blasts (CD31^hi^Ly6C^−^), myeloid blasts (CD31^+^Ly6C^+^) and MNs (CD31^−^Ly6C^hi^) have different capacity to differentiate into OCLs (46). BM myeloid blasts that express higher CD115 levels differentiate more efficiently and faster than early blats and MNs (46). Indeed, while c-kit is down regulated during murine OCL differentiation, CD115 expression is maintained (44), which is essential for osteoclastogenesis since binding of CD115 to its ligand M-CSF increases the expression of RANK, allowing further differentiation into OCLs under RANKL stimulation (41). Moreover, analysis using BrDU incorporation in mice revealed that quiescent CD115^+^ RANK^+^ progenitors represent committed OCL progenitors able to circulate and settle down in the bone (47, 48).

Interestingly, the origin of OCLs from BM HSCs and downstream progenitors appears to be restricted to adults. This was very recently demonstrated by Jacome-Galarza et al. who depleted RANK or CD115 expression in BM-HSC progenitors (using *Csf1r*^cre^ mice) or in BM-HSC and erythro-myeloid progenitors (EMP) (using *Flt3*^cre^ mice). Depletion of these genes in embryonic EMP resulted in osteopetrosis in newborns, whereas only adults were affected by specific depletion in HSC progenitors (38). This study elegantly demonstrated for the first time that OCLs involved in fetal bone formation and tooth eruption are originating from the same progenitors as tissue Mϕs and differ thereby from OCLs arising in adults (Figures 2A,B) (38, 49).

Therefore, the interplay between OCLs and other monocytic cells appears much more puzzling than the existence of a common BM progenitor. *In vitro*, OCLs can be generated in the presence of RANKL and M-CSF from hematopoietic cells originating from many tissues. BM in mouse and blood in human are major sources of OCL progenitors, but OCLs can also be obtained from hematopoietic cells from the liver, spleen, thymus and lymph nodes, suggesting a high heterogeneity of potential OCL precursors (3, 50–54). Indeed, in addition to their differentiation from BM progenitors, OCLs can also arise from cells already engaged in the MN or DC pathways (3, 13, 14, 55, 56). As described below, this process is associated with pathological conditions related to bone destruction making OCL differentiation more complex and dynamic than expected. Thus, there is a large variability in OCL precursor cells depending on the signals they receive from their normal or pathological environment. These observations strongly support that OCLs do not represent a single and homogeneous population but that they display the same heterogeneity in their origin and phenotype as other monocytic cells (Figure 2C).

### Osteoclasts and Monocytes

Monocytes are innate immune cells characterized by a great level of plasticity and found in all tissues. Depending on the environmental cues, they can differentiate into DCs, Mϕs or OCLs. Being involved in both inflammation and bone resorption, MNs represent key regulators of bone tissue homeostasis. Moreover, they are heterogeneous and comprise several subtypes already committed to different functions. Knowledge on MNs has evolved a lot over the past decade thanks to new technologies such as fate mapping and single-cell RNA sequencing (scRNAseq). Historically, they were considered as an intermediate status in the peripheral circulation, between myeloid precursors from the BM and Mϕs in tissues. Now, we know that certain tissue-resident Mϕs are generated independently of MNs during early phases of embryogenesis and that fetal MNs derive from multipotent erythro-myeloid progenitors. Later and throughout life, MNs are generated from the HSC-derived hematopoiesis (49, 57), as described for OCLs (38). In healthy conditions, MNs are present in central (BM) and peripheral (spleen) reservoirs and engrafted into certain resident macrophage pools. They are not only involved in the repopulation of tissue Mϕ and DC compartments but also contribute to the establishment and resolution of local inflammatory reactions and participate in the innate immune surveillance of the organism (58). Under pathological conditions, they are rapidly mobilized in large numbers and recruited to the inflamed tissue where they display both inflammatory and pro-resolving properties to allow tissue repair. This last step must be transient. If monocyte numbers in tissues are not properly regulated and persist overtime, their actions become pathogenic for targeted tissues (59). In particular, massive recruitment of MNs in the BM is associated with increased OCL formation and bone destruction (56, 60).

Two main subsets of MNs exist both in mouse and human (61, 62). In mice, the “classical” monocytes are characterized by the combination of specific surface markers Ly6C^high^CCR2^+^CX3CR1^low^CD62L^+^Gr1^+^, and were previously named inflammatory monocytes because they can differentiate into inflammatory Mϕs and inflammatory DCs (63, 64). The “non-classical” MNs are characterized by the Ly6C^low^CCR2^low^CX3CR1^+^CD62L^−^Gr1^−^ surface markers and are also named patrolling MNs because they survey endothelial cells and surrounding tissues for damage or viral infection. Their human counterparts are CD14^+^CD16^−^ and CD14^low^CD16^+^, respectively (61, 65). A recent scRNAseq analysis of human blood confirmed that classical and non-classical MN subsets represent two distinct clusters (66). Several genetic mouse models of deletion of transcription factors, cytokines and chemokines, together or not with acute or chronic inflammatory challenges, have been used to delineate functional heterogeneity of the Ly6C^high^ and Ly6C^low^ monocyte subsets *in vivo*. Only few studies however addressed their respective capacity to differentiate into OCLs.

It is now well established that mature mouse Ly6C^high^ MNs differentiate into the BM from unipotent common monocyte progenitors, with an intermediate Ly6C^high^CXCR4^+^ pre-monocytic step (67). Under steady state conditions, mature Ly6C^high^ MNs constantly egress from the BM in a CCR2-dependent manner (68) following circadian oscillations (69) and circulate for 1 day in the blood. Then, a minority (~1%) of these MNs convert into non classical Ly6C^low^ MNs that have longer circulating lifespans (~7 days), while the vast majority (~99%) of Ly6C^high^ MNs leave the circulation and replenish specific pools of tissue resident Mϕs (70–72). For specific peripheral inflammatory responses to infections or tissue damages, the fate of mouse Ly6C^high^ MNs is identical. Differences rely on the speed and amplitude of the mobilization from BM and spleen to target sites, the kinetic being faster and larger numbers being produced, leading to blood monocytosis as early as 4 h after endotoxin challenge (73). Additionally, differences also rely on the fraction of blood monocyte-derived Mϕs that replenishes tissue-resident Mϕs after recruitment (71). Mouse Ly6C^high^ MNs are precursors of longer-lived Ly6C^low^ MNs that express higher levels of CX3CR1 (74) and continuously patrol the luminal side of the vasculature in a CX3CR1 and LFA-1/ICAM1-dependent crawling manner (58). Under physiological conditions, Ly6C^low^ MNs are the endothelium housekeepers, playing a key role to control endothelium integrity by scavenging luminal microparticles, recruiting neutrophils for focal necrosis of endothelial cells, phagocytizing cellular debris (75). Upon bacterial infection, Ly6C^low^ MNs secrete IL-10 and are recruited to tissue where they more likely differentiate into alternatively activated Mϕs, contributing to tissue repair. Ly6C^low^ monocytes can also promote tolerance to self-antigens contained in apoptotic cells through a PD-L1-dependent mechanism and thanks to their high capacity to phagocyte apoptotic cells *in vivo* (76). Overall, the multiple capacities of both MN subsets to differentiate into either regulatory or inflammatory mature Mϕs or DCs depend on the inflammatory signal and tissue microenvironment. Interestingly, both mouse MN subsets can go back to the BM thanks to a CXCR4-dependent signal (67). The respective role of Ly6C^high^ and Ly6C^low^ MNs on bone turnover remain yet to be established.

Since MNs constitute a source of OCLs, it is expected that both MN subsets display OCL differentiation potential. Although the culture conditions used *in vitro* to monitor OCL differentiation diverge between studies, it appears that mouse OCLs develop from BM CD11b^−/low^Ly6C^high^ monocytic progenitors (as described above) and from blood CD11b^high^Ly6C^high^ MNs. In the BM, CD11b^−/low^Ly6C^high^ monocytic progenitors are more prone than CD11b^+^ MNs to differentiate into OCLs (43) because of the negative role of CD11b and β2-integrin signaling on OCL differentiation (77). *In vitro* comparative studies based on BM treatment with various cytokines demonstrated that Ly6C^high^ MNs were far more efficient than Ly6C^low^ monocytes to differentiate into mature OCLs (78). Importantly, the BM CD11b^−/low^Ly6C^high^ population also displays an OCL differentiation capacity *in vivo* and is expanded in inflammatory arthritis models (79). In particular, the CX3CR1^+^ fraction of these cells is highly enriched in OCL precursors (79). In-depth phenotypic characterization allowed to further dissect CD11b^−/low^Ly6C^high^ cells into three different populations with high osteoclastogenic potential based on the expression of the phenotypic marker CD117 (c-Kit) (43).

In the blood, the mouse Ly6C^high^ MN subset also represents the major precursor cell population of OCLs (Figure 2C). Indeed, *in vitro* Ly6C^high^ MNs are more efficient than the Ly6C^low^ subset to differentiate into TRAcP positive cells (55). In the context of inflammatory arthritis, disease severity is associated with Ly6C^high^ blood monocytosis, and Ly6C^high^ MNs more specifically migrate to the inflamed joints and contribute to bone erosion due to their excessive differentiation into OCLs (56). Importantly, *in vivo* delivery of therapeutic molecules to Ly6C^high^ MNs, but not to Ly6C^low^ MNs, markedly interferes with pathogenic bone erosion in experimental arthritis, suggesting that the classical subset represents a candidate cell target for anti-osteoclastogenic strategy design (56). In human, OCLs generated from peripheral blood MNs originate from the classical CD14^+^CD16^−^ subset, and not from the CD16^+^ subset, in an integrin β3-dependent manner (80, 81). Later studies refined this view showing that while the different human MN subsets can differentiate into OCLs when cultured on plastic, OCLs are predominantly formed from classical MNs when cultured on bone slices (82). In the context of inflammatory bone diseases, the distribution of MN subsets is skewed toward a higher proportion of CD14^+^CD16^+^ MNs (83). Interestingly, it seems that in these pathological conditions, CD14^+^CD16^+^ MNs are more prone to differentiate into OCLs than in healthy conditions, as demonstrated in psoriatic arthritis patients (84).

The origin of OCLs from MN progenitors or MNs is likely to be dictated by the BM cell environment. Indeed, monocytic OCL precursors show different expression level of cytokines/growth factors leading to different effect of inflammatory cytokines. In human, among the three MN subsets, only the CD14^high^ CD16^+^ intermediate subset responds to IL-17 by forming larger OCLs having higher resorption capacity than in absence of this cytokine (82). In mouse, BM CD31^high^Ly6C^−^ early blasts, CD31^+^Ly6C^+^ myeloid blasts and CD31^−^Ly6C^high^ MNs are differently affected by their environment. When assessing life span, IL-1β enhance OCL formation especially of myeloid blasts, which have rapidly formed and have a short life span, while OCLs derived from CD31^−^Ly6C^high^ MNs are formed a later stage and have a longer life span (85). Remarkably, M-CSF pretreatment of myeloid blasts or TNFα pretreatment of MNs before addition of RANKL inhibit osteoclast formation from CD31^−^Ly6C^high^ MNs but not from early blast or myeloid blast despite the expression of RANK (86, 87). These cells were able to regain their osteoclastogenesis capacity when cultured on bone slices, revealing the importance of the bone attachment and signaling in OCL differentiation. This is further supported by the finding that collagen specific motifs are ligands for OSCAR, a costimulatory receptor induced by RANKL and essential for OCL differentiation (88).

### Osteoclasts and Dendritic Cells

Dendritic cells have been identified by Steinman and Cohn 45 years ago (89). More than through their phenotype and surface marker expression, DCs are also defined by their functional specificity. Being located in most tissues where they represent 1–5% of the hematopoietic cells, they act as sentinels of the immune system capturing and processing antigens and instructing adaptive immune cells (84). Contrasting with MNs and Mϕs, they have the unique capacity to migrate to the T cell zones of lymphoid organs where they present or cross-present antigens thanks to their expression of major histocompatibility complexes (MHC)-I and -II and activate naive T cells.

As MNs, DCs represent a heterogeneous population of cells. In mouse, DCs that reside in lymphoid organs are composed of CD8^+^ and CD8^−^ conventional/classical DCs (cDC) and IFNα-producing plasmacytoid DCs (pDCs). Equivalent subsets are also present in human, namely CD1c^+^ (BDCA1) and CD141^+^ (BDCA3) cDCs and CD303^+^ (BDCA2) pDCs (90, 91). Plasmacytoid DCs have the capacity to produce high amounts of IFNα in response to viral and foreign nucleic acids stimulation and to prime naive T cells against viral antigens (92).

In murine non-lymphoid tissues, the two main cDC subsets are CD11b^+^ and CD103^+^CD11b^−^ (90). These cDCs have a tremendous capacity to permanently sense their environment and uptake antigens in tissues and blood. They express high levels of MHC complexes and the machinery to process and present antigens, they have very high migratory capacity to the lymph nodes mainly governed by the chemokine receptor CCR7 (93) and they are highly efficient in naive T cell activation and polarization. By driving T cell differentiation toward different T helper (Th) subsets or regulatory T (Treg) cells depending on their activation and the cytokine they produce, DCs have the capacity to induce immune responses against foreign antigens or to stimulate self-antigen tolerance (94, 95).

The majority of splenic and lymphoid organ DCs are renewed from BM progenitors (96, 97) and Flt3L play a major role in their homeostasis (98, 99). In human and mouse, cDCs and pDCs arise from BM Flt3^+^CD115^+^c-kit^low/int^ common dendritic progenitors (CDP), downstream from progenitors common to MNs, DCs, Mϕs and OCLs (39, 97, 100, 101). DCs precursors egress from the BM and migrate to lymphoid organs and tissue to differentiate into immature cDCs (102). In contrast, pDCs are generated in the BM and then disseminate to lymphoid tissues (102). Upon inflammation or infection, inflammatory DCs are transiently generated from classical Ly6C^high^ MNs that are recruited into inflamed tissues, and drive T cell activation in the draining lymph nodes (63, 103–105). Contrasting with cDCs, inflammatory DCs are not depend on Flt3L since they are generated in Flt3L^−/−^ mice (106). *In vitro*, inflammatory DCs can be generated from MNs or BM cells in the presence of GM-CSF and IL-4 (105, 107). However, *in vivo* studies in knockout mice identified M-CSF receptor (CD115) as a major factor controlling their development (108). In addition to naive T cells in lymphoid organs, inflammatory DCs can also activate T cells in the inflamed tissues, in particular memory T cells (109). This function appears to be dependent on the antigen dose and the severity of inflammation (105, 106).

Over the past decade, different groups have reported on the capacity of DCs to give rise to OCLs, as described below. This was surprising as DCs are considered as fully differentiated cells and have been described to have a short life (~1.5 to 3 days) (110, 111). However, more recent data revealed that about 5% of DCs still maintain a proliferation capacity (96, 112–114). DC survival is increased in the presence of lymphotoxin LTα1β2 (112) and RANKL (115, 116). Moreover, mature DCs can undergo further proliferation and differentiation into regulatory DCs when they are in contact with splenic stromal cells (117). These observations support a high plasticity in the fate of DCs depending on their environment.

In regard of the huge number of publications dealing with DCs in secondary organs or even in tissues, very few are focused on mature BM DCs. As in other tissues, DCs represent a small population of BM cells (1–2%) expressing high levels of CD103 (118, 119). They are mainly located in the perivascular region where they form clusters, interact with T cells and provide survival signals to B cells (119). As BM is a major reservoir of memory T cells, the presence of DCs in this organ is thought to contribute to reactivation of these memory T cells (120, 121). In contrast, naive T cell activation is supposed to be restricted to secondary lymphoid organs. However, and surprisingly, BM DCs also have the capacity to present and cross-present antigens to naive CD4^+^ and CD8^+^ T cells and to activate them as efficiently as DCs from lymphoid organs (122, 123). This T cell-priming activity of BM DCs is independent of spleen and lymph nodes as it was also observed in mice lacking these organs (122). These observations revealed that the BM is not just a primary lymphoid organ but that it shares some features with secondary lymphoid organs.

The first demonstration of the differentiation of OCLs from DCs was evidenced using human immature DCs generated *in vitro* from blood CD14^+^ CD16^low/−^ MNs. In response to M-CSF and RANKL, these cells differentiate as efficiently as MNs into bone-resorbing OCLs (13). Human MN-derived DCs differentiate faster than MNs and form OCLs having more nuclei than from MNs (13). This was later confirmed with murine DCs (Figure 2C). Splenic cDCs were shown to efficiently differentiate into OCLs in the presence of RANKL and M-CSF and among them the CD8^−^ subpopulation is the more efficient OCL precursor, whereas OCL differentiation from pDCs is less efficient and takes longer than from cDCs (14). More importantly, this differentiation pathway was also demonstrated *in vivo* using osteopetrotic *oc/oc* mice (14, 15) in which OCLs are abundant but inactive (50). Transfer of cDCs from normal mice into *oc/oc* mice restored bone resorption and improved mice survival demonstrating that OCLs can differentiate from DCs *in vivo* and that osteopetrosis can be treated not only by hematopoietic stem cell transplantation but also by DCs infusion (14, 15). Interestingly, DC-derived OCLs and MN-derived OCLs show an equivalent expression of the main OCL markers (124) and both have the same capacity to resorb bone (13, 14) demonstrating that both populations correspond to *bona fide* OCLs.

As described above for MNs, the differentiation of OCLs from DCs is modulated by their environment. First, this differentiation has been confirmed *in vitro* and *in vivo* in a number of different pathologies related to inflammation or cancer, but never in a healthy context (125) (Table 1). Indeed, *in vitro*, TNFα, IL-1α, IL-17 or synovial fluid from arthritic patients enhance the differentiation of human MN-derived DCs into OCLs (13, 19). *In vivo*, the presence of Th17 cells or high levels of RANKL are required to induce the DC-to-OCL differentiation as demonstrated using osteopetrotic *oc/oc* mice or in the context of multiple myeloma (14, 21).

From all these observations, it appears that while adult OCLs differentiate from BM progenitors in steady state, they can also arise from differentiated MNs and DCs in pathological conditions. The origin of OCLs is therefore depending on the developmental stage, the BM environment and the BM recruitment of MNs or DCs. Moreover, OCLs are able to constantly incorporate new nuclei and split off other groups of nuclei both *in vitro* (126) and *in vivo*^1^ (38). Parabiosis experiments between Csf1r^cre^;Rosa26^LSL−YFP^ and Csf1r^cre^;Rosa26^LSL−tdTomato^ mice revealed that 0.5–2% of OCLs acquire new nuclei per day and can persist several weeks *in vivo* (38). Thus, depending on the cells that are present at their vicinity, it is very likely that OCLs are formed by a mix of different OCL progenitors instead of originating from a pure progenitor population, reflecting a high flexibility and the capacity to rapidly adapt to different pathological circumstances.

## The Immune Function of Osteoclasts

A number of studies have been conducted on the regulation of OCLs by T cells under inflammatory conditions. Inflammation and bone destruction were observed to occur side by side, as shown for example for rheumatoid arthritis (RA) (127–129), periodontitis (130–133) or inflammatory bowel disease (IBD) (134, 135). During inflammatory states, it has been described that OCL progenitors respond to several interleukins produced by activated CD4^+^ T cells such as RANKL, TNFα, and IL-17, including in human (52, 116, 136–139). Among CD4^+^ T cells, only Th17 cells have been shown to induce or enhance OCL differentiation (56, 129, 131, 134–136). During inflammatory conditions, IL-17-expressing Th17 cells were also associated with increased bone destruction and osteoclastogenesis by up regulating RANK in OCL progenitors and by inducing RANKL expression in osteoblasts (60, 137, 140–142). Moreover, they increase the expression of monocyte-attractant chemokines such as MCP1 and MIP1α in osteoblasts and induce *in vivo* the recruitment of OCL monocytic progenitors in the BM (60). In contrast, other T cell-derived cytokines such as IFNγ, IL-4, IL-10, IL-12, and IL-18 as well as regulatory T cells were reported to have a negative effect on osteoclastogenesis (137, 143–146).

Besides numerous studies investigating the potential effect of T cells on OCLs, the reciprocal contribution of OCLs to immune modulation and the understanding of immune responses caused directly by OCLs, as for instance as antigen-presenting cells, remained undiscovered for a long time. Genome-wide expression analysis strengthened the idea that OCLs have an immune function. Indeed, comparative micro-array transcriptomic analysis revealed that *in vitro* differentiated human OCLs are transcriptionally closer to DCs than to MNs (124). Furthermore, during their *in vitro* differentiation process, murine OCLs arising from CD11b^+^ BM cells up regulate sets of genes involved phagocytosis and immune responses (147). More recently, studies emphasized OCLs as true antigen-presenting cells to regulate and control T cell activation as well as their ability to initiate T cell responses in an antigen-dependent manner (18, 32, 33). Considering OCLs as cells having an immune function besides the classical bone resorption activity is a very new concept that is elaborated in more detail in the following sections.

### Bone Resorption and Phagocytosis

**Figure 3:**
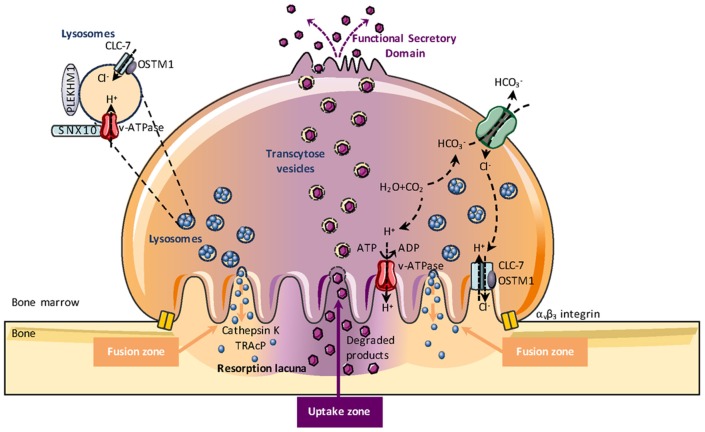
Osteoclast-mediated bone resorption. Bone degradation requires the extracellular secretion of lysosomal enzymes, such as TRAcP (Tartrate-resistant acid phosphatase) and cathepsin K into the resorption lacuna. This exocytosis of lysosomes occurs in the peripheral region of the ruffled border (fusion zone) and is guided by PLEKHM1, OSTM1, and SNX10. Proton pumps (v-ATPase) and the CLC7-antiporter are relocated at the ruffled border plasma membrane of bone-resorbing OCLs and mediate extracellular acidification for bone demineralization during bone resorption. The v-ATPase transports protons obtained by carbohydrase-dependent hydration from CO_2_ to H_2_CO_3_ into the resorption lacuna while HC${\text{O}}_{3}^{+}$ is excreted via the Cl^−^/HCO_3_ exchanger. Molecular degradation products generated during bone resorption are released through the central region of the ruffled border (uptake zone). Through transcytosis trading, vacuoles are able to cross the OCL cytoplasm and exit the OCL from the so-called “functional secretory zone” at the basolateral plasma membrane into the vascular space.

The best-known OCL function is bone resorption and OCLs are regarded as professional phagocytes specially adapted to resorb bone. Contrasting with classical phagocytosis in which internalized material is degraded in intracellular endo-lysosomal vesicles, this unique property of OCLs is accomplished through an extracellular mechanism that makes the OCL a peculiar “giant macrophage” with molecular machinery distinct from any other cell type. After mature OCLs have polarized onto the bone surface, a tight and dynamic seal segregates the underneath extracellular space (later becoming the resorption lacuna) from the rest of the extracellular bone marrow space (148). A massive extracellular proton and enzyme secretion occurs through exocytosis of lysosomes at the most peripheral area of the ruffled border (Figure 3) (4, 149). Lysosomal membranes are inserted into this plasma membrane domain, introducing here the vacuolar H^+^-ATPase (proton pump) and the ClC7 2Cl^−^/1H^+^ antiporter (chloride channel type 7), while the acidic hydrolases are released into the resorption lacuna microenvironment. Thus, the resorption lacuna has a composition very similar to the one of intracellular endosomes, with low pH, high calcium concentration and abundance of acidic hydrolyses (150, 151). The ruffled border shares membrane characteristics of late endosomes (152). When bone resorption is completed, the resorption lacuna is filled mainly with calcium and phosphate ions as well as small collagen type I fragments. Dynamic studies showed that the inner uptake domain of the ruffled border is specialized in the clathrin-mediated endocytosis of the degraded bone matrix (149, 153, 154) that allows internalization of nanosize particles (155). Then, by a small GTPase-dependent transcytosis trafficking, vacuoles cross the osteoclast cytoplasm to reach the functional secretory zone opposite to the ruffled border (153). Through this domain, the degraded bone products are released in the extracellular microenvironment and taken up by the vascular stream. Finally, although the OCL transcytosis function has been elegantly demonstrated by various groups, this does not exclude that degraded matrix components could leak out from the resorption lacuna when the tight seal of this environment is loosened and the OCL moves away from the previous resorption site to reach a new site where it re-polarizes and starts a new resorption cycle (156).

Beside their specialization in bone resorption, OCLs display the classical phagocytic properties shared by monocytic cells. Phagocytosis is a key form of endocytosis for the clearance of large particles (with few μm range) such as pathogens, microorganisms and abnormal or dead cells and it is essential for tissue repair and modeling, and for fighting against infection (31, 155). Phagocytes express a set phagocytic receptors belonging to non-opsonic receptors (such as scavenger receptors or c-lectin and lectin-like receptors) (157, 158) and to opsonic receptors (such as complement receptors and Fc receptors) (159, 160) able to recognize different microbial components and altered cells. This binding creates a phagocytic synapse and invagination of the cell membrane, activates signaling pathways, and leads to the engulfment of the particles in phagosomes, a process associated with a high dynamic cytoskeleton remodeling (161). The phagosome undergoes maturation and fuses with lysosomes to form a phagolysosome having the capacity to degrade the ingested particle (31, 161). As the resorption lacuna, the phagolysosome is characterized by a low pH (4.5 to 5) maintained by V-ATPases and ClC family antiporters, and contains hydrolytic enzymes, bactericidal proteins, cationic peptides, and oxidants (162). Depending on the nature of the phagocytic cells, degradation of the ingested particles is different: while proteolysis is extensive in macrophages, it is more partial in DCs to allow the degraded peptides to associate with the MHC complexes for antigen presentation (31).

As other monocytic cells, OCL express a number of factors that play crucial roles in phagocytosis (30, 147). In OCLs, phagocytosis may provide an additional mechanism participating in degradation of calcium-phosphate (CaP) materials together with resorption (29, 163). Indeed, ultrastructural microscopy analysis revealed that OCLs are able to engulf in endophagosomes CaP crystals that undergo intracellular degradation instead of degradation in the resorption lacuna (29). They also internalize other particles such as latex beads or polymethylmethacrylate particles while they maintain their bone resorbing activity (164). This mechanism probably further increases the capacity of OCLs to degrade large particles that are difficult to resorb through classical bone resorption associated with clathrin-dependent endocytosis (29, 165). It can also be involved in implant loosening where phagocytosis of wear particles derived from implants is associated with bone destruction (29, 164).

Interestingly, phagocytosis can occur in glass-seeded OCLs that are not polarized, showing that this process is not necessarily associated with bone resorption (166). This has been demonstrated for phagocytosis of damaged cells. *In vitro*, OCLs have the capacity to recognize and engulf apoptotic bone cells, such as chondrocytes and osteocytes (30, 167, 168) but also other cell types as shown for instance for glutaraldehyde-fixed red blood cells (169) and apoptotic thymocytes, as efficiently as macrophages (30). *In vivo*, this mechanism may participate in regulating inflammation and autoimmunity by clearing apoptotic bone cells that are embedded in a matrix that only OCLs have the unique capacity to resorb (30). These data strongly support that bone resorption is not the sole function of OCLs and that these cells are participating more largely to the immune regulation in the BM.

### Osteoclasts and Antigen Presentation

Beside this unique resorption function in bone homeostasis and repair, the close relationship of OCLs with MNs, Mϕs, and DCs raises the question of the potential immune function of OCLs. The fact that OCLs respond to immune signals and that the bone and the immune system are in close proximity to each other and interact through cellular and molecular exchanges led to the conclusion that OCLs are more than just simple bone resorbing cells (36, 170–172). Indeed, it would not be surprising that OCLs maintain and share similar functions to monocytic cells, in particular DCs that are known to be professional antigen presenting cells (APCs).

Professional APCs are defined as immune cells able to process and present antigens via major histocompatibility complex (MHC), and activate naive T cells through interaction between MHCs and T cell receptors (TCRs), costimulatory signals and cytokine production. Antigens are recognized by T cells in the form of short peptides that have been processed and presented on the APC surface bound to MHC class I or MHC class II molecules (173–175). While MHC-I are ubiquitously expressed on all nucleated cells to present intracellular antigens, MHC-II molecules are constitutively expressed on professional APCs to present exogenous antigens (176, 177). However, only cDCs have the feature to cross-present antigens through MHC-I complexes to prime CD8^+^ T cells. TCR on CD8^+^ and CD4^+^ T cells binds to the MHC-I and MHC-II complexes, respectively, in order to initiate naive T cell activation and to trigger TCR signaling (178, 179). A crucial step in T cell activation is mediated by co-stimulatory molecules such as CD80 (B7.1) and CD86 (B7.2) that determine the functional outcome of the TCR signaling (180, 181). Subsequently, APCs start to secrete cytokines that control the differentiation of activated T cells into effector T cell subsets such as Th1, Th2, or Th17 subsets to promote inflammatory responses or regulatory T cells that regulate immunosuppressive responses (181–184).

**Figure 4:**
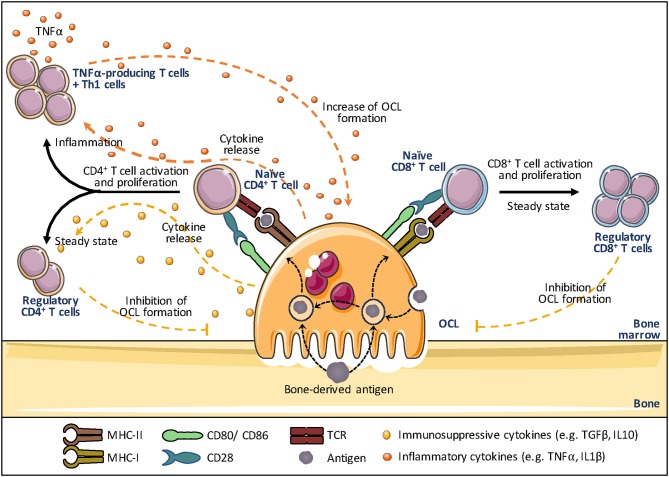
Osteoclasts and antigen presentation. Osteoclasts (OCLs) function as antigen presenting cells (APCs). OCLs process and present antigens (coming from the bone through bone resorption or the bone marrow) in the form of short peptides bound to major histocompatibility complex (MHC)-I or MHC-II molecules. The T cell receptor (TCR) on CD8^+^ and CD4^+^ T cells binds to the MHC-I and MHC-II complex, respectively, to initiate T cell activation and to trigger TCR signaling. A key step in T cell activation represents the T cell binding to co-stimulatory molecules (e.g. CD80 and CD86 on OCLs and CD28 on T cells), as they determine the functional outcome of the T cell receptor signaling. Subsequently, OCLs start secreting cytokines that control the differentiation of activated T cells. In steady state, OCLs produce mainly IL-10 and TGFβ and induce regulatory T cells (Treg) that are responsible for immunosuppressive responses and inhibit OCL differentiation, creating a negative feedback down regulating both inflammation and bone resorption. In contrast, in inflammation, OCLs induce effector TNFα-producing T cells that promote inflammation and may stimulate osteoclastogenesis thanks to their TNFα production. The effect of OCLs on CD8^+^ T cells in inflammatory conditions has not been explored yet.

In addition to DCs, professional APCs also comprise B cells and Mϕs that have all constitutive expression of MHC-II (173, 185, 186). Other immune cells (including basophils, innate lymphoid cells, neutrophils, mast cells) and non-hematopoietic cells (including endothelial cells, mesenchymal stromal, epithelial cells) have been reported to upregulate MHC-II upon stimulation (187). But the absence of constitutive MHC-II expression, the lack of phagocytic function and a less evident capacity to prime naive T cells in an antigen-dependent manner make these cells atypical APCs (187). Rivollier et al. first showed that human OCLs *in vitro* differentiated from blood MN-derived DCs constitutively express HLA-DR and CD86 (13). This was further confirmed in murine OCLs differentiated *in vitro* from MNs or DCs (18), but also *in vivo* in bone marrow OCLs (18). The assumption that mature OCLs could act as APCs was equally reinforced by others (Figure 4). Li et al. observed that similar to DCs, human OCLs express MHC-I and II as well as the co-stimulatory molecules CD80, CD86, and CD40 (33). They also showed that OCLs are able to engulf soluble antigens and to present them on their cell surface demonstrating that OCLs can function as APCs. Additional cytokine assays confirmed that OCLs, similar to DCs, express IL-1β, IL-6, IL-10, transforming growth factor-beta (TGF-β), and TNFα further indicating that they meet the demands as APCs (18, 33). Grassi et al. strengthened this hypothesis by showing that OCLs derived from human monocytic precursors expressed MHC-II and upregulated the expression of CD80 and CD40 during their differentiation (34). Ibáñez et al. have explored this function in murine OCLs using DQ-OVA, a BODIPY-conjugated form of ovalbumin that becomes fluorescent after endolysosomal degradation. They showed that OCLs differentiated *in vitro* from MNs and from DCs both express MHC-II and costimulatory molecules and are able to process and present ovalbumin at least as efficiently as DCs (18). Furthermore, using I-A^b^β-GFP knock-in mice that express a fusion protein between GFP and the I-A^b^β subunit of the MHC-II complex, they demonstrated that OCLs constitutively express MHC-II *in vivo* (18). Lastly, OCLs were also shown to cross-present antigens via MHC-I, a feature considered to be specific for cDCs (32). Interestingly, OCLs process and present antigens even when they are cultured on plastic or when they are not able to resorb bone as shown for OCs from *oc/oc* mice (18). In addition, OCLs that process antigens still retain their bone-resorbing capacity, revealing that antigen presentation and bone resorption are independent functions in OCLs (18). These findings expanded the field of osteoimmunology by directly connecting OCLs to the immune system.

### Osteoclasts and T Cell Activation

While T cell activation is usually assumed to take place in secondary lymphoid organs such as the spleen and lymph nodes, naive T cell priming, activation and polarization into effector T cells as well as memory T cell reactivation can also occur in the BM, as described above (120–123). BM T cells represent ~3–8% of total BM cells and, compared with blood, the BM CD4/CD8 ratio is characteristically decreased (188, 189). Furthermore, in comparison to other lymphoid organs, memory CD4^+^ T cells specific for previously encountered antigens represent the vast majority of T cells observed in the BM, which includes central memory as well as effector memory T cells (190–192). Therefore, the BM microenvironment is often described as a major reservoir for memory lymphocytes (121, 193–195). Interestingly, T cells are often observed in the close vicinity of OCLs (34, 196–198) or adherent to OCLs (198). Furthermore, OCLs were shown to express chemokines involved in T cell chemotaxis and are able to attract CD4^+^ and CD8^+^ T cells *in vitro* as efficiently as DCs (18, 32, 34). Together with their APC function, this supports a contribution of OCLs to the activation of T cells in the bone marrow (Figure 4).

Based on transcriptomic analysis showing a high expression of genes related to antigen presentation in mature OCLs (147), Seifert et al. addressed the capacity of OCLs generated from murine BM cells to activate CD8^+^ T cells (32). Using an antigen-specific system, they reported that OCLs induce CD8^+^ T cell proliferation and activation by antigen cross-presentation (32), a function unique to DCs that participate in anti-infectious responses and self-tolerance (199). Interestingly, these OCL-primed CD8^+^ T cells expressed FoxP3, the master gene of regulatory T cells, and have a suppressive effect attested by their capacity to reduce the proliferation of other naive responder CD8^+^ T cells induced by DCs (32). This study clearly revealed for the first time that OCLs are not only regulated by T cells but can initiate T cell responses themselves, creating a feedback control loop. Indeed, Buchwald et al. (200) showed that the CD8^+^ Treg cells induced by OCLs are in turn able to suppress the resorptive function of OCLs as well as their differentiation.

**Figure 5:**
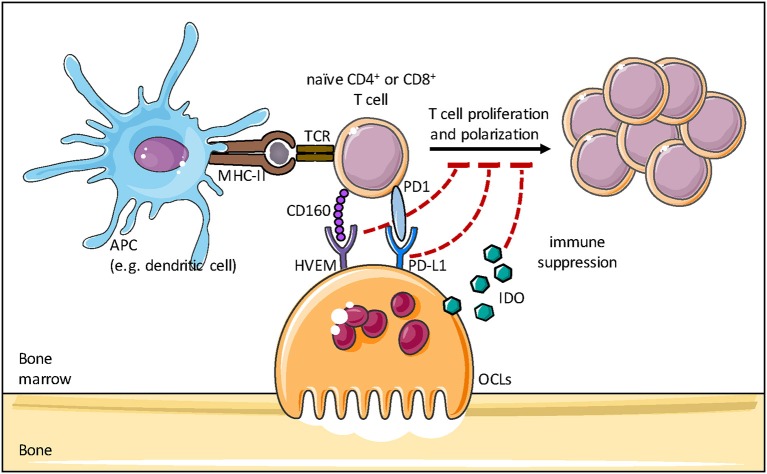
Osteoclasts and immune modulation. Osteoclasts are able to induce an immunosuppressive microenvironment by upregulating inhibitory checkpoint molecules such as programmed death ligand 1 (PD-L1), herpes virus entry mediator (HVEM) and Indoleamine 2,3-dioxygenase (IDO), thereby inhibiting the proliferation and polarization of naive T cells induced by antigen presenting cells (APCs).

Besides these findings, OCLs are also able to interact with naive CD4^+^ T cells. Using a reliable procedure to sort pure OCLs (18, 51), Ibanez et al. (18) demonstrated that OCLs from the BM of healthy mice induced FoxP3^+^ CD4^+^ T cells in an antigen-specific manner. These cells were shown to inhibit the activation of CD4^+^ T cells, confirming that they are immunosuppressive CD4^+^ Treg cells (18). This capacity was confirmed in human OCLs using T cells from tetanus toxoid (TT)-immunized healthy donors (33). OCLs pulsed with TT activate CD4^+^ T cells that produce high levels of the immunosuppressive cytokines IL-10 and TGF-β, evoking suppressive T cells (33). OCL-induced immunosuppression has also recently been reported in association with hematologic malignancies (35, 36, 201). In the context of multiple myeloma (MM), OCLs were shown to play a crucial role in the induction of an immunosuppressive microenvironment by upregulating inhibitory checkpoint molecules such as programmed death ligand 1 (PD-L1), CD200 and Galectin-9 as well as immunosuppressive cytokines (Figure 5). In addition, OCLs protect MM cells against T cell-mediated cytotoxicity through inhibition of CD4^+^ and CD8^+^ T cells and thereby support the development of the malignancy (35).

However, OCLs are not restricted to immunosuppressive function. Indeed, while OCLs derived from healthy mice induce immunosuppressive CD4^+^ Treg cells, those derived from mice under inflammatory conditions induced TNFα-producing CD4^+^ T cells, as shown in the context of inflammatory bowel disease (18), a chronic inflammatory disease associated with increased OCL differentiation and severe bone destruction (60) (Figure 4). As OCLs from healthy mice, MN-derived OCLs induce CD4^+^ Treg cells and express higher levels of the immunosuppressive cytokine IL-10 than DC-derived OCLs (18). In contrast, as OCLs derived from mice affected by chronic inflammation, DC-derived OCLs very efficiently induce differentiation of TNFα^+^ CD4^+^ T cells and express higher levels of inflammatory cytokines (TNFα, IL-1β, IL-6) than MN-derived OCLs (18). These data unveiled for the first time the existence of different OCL subsets having opposite effects on the immune system depending on their context and cell origin. Searching for markers specific for murine OCL subsets, Ibanez et al. identified by flow cytometry the fractalkine receptor CX3CR1 as the first marker specifically expressed in inflammatory OCLs. Admittedly, only about 20% of inflammatory OCLs are positive for CX3CR1 (18) and the role of CX3CR1 in these OCLs as well as the function of CX3CR1^+^ and CX3CR1^−^ inflammatory OCLs have not yet been addressed.

### Physiological and Clinical Relevance of the Immune Function of Osteoclasts

In the end, the question remains what the biological relevance of antigen presentation by OCLs is. Comparable to APCs, OCLs mainly use clathrin-mediated endocytosis and micropinocytosis to internalize bone degradation products (179, 202). Thus, the physiological significance of the immune function of OCLs may be associated with the sustained release of self-peptides from bone resorption. These self-peptides can be presented by OCLs to inhibit self-responses by producing immunosuppressive cytokines and inducing suppressive Treg cells (18, 32, 200). In addition, OCLs have the ability to engulf and present antigens that are not coming from bone resorption (18, 34). This may also include antigens originating from the periphery such as blood-borne antigens (122, 123) or antigens carried by circulating DCs, neutrophils and B cells (120, 203) that can be presented directly on MHC-II molecules. Interestingly, the proportion of Treg cells among T cells in the BM is higher than in other tissues (204, 205) and they provide the BM with an immune privilege that is required for the maintenance of hematopoietic stem/progenitor cells (205). They also down regulate OCL differentiation participating thereby to the control of bone homeostasis (206–208). Thus, OCLs could therefore be regarded as BM resident APCs participating to maintain the BM immune tolerance under physiological conditions.

Contrary to this, OCL subsets generated under inflammatory conditions are devoid of the capacity to induce Treg cells but instead they induce effector CD4^+^ T cells that produce TNFα, an inflammatory cytokine that stimulates osteoclastogenesis and promotes inflammation (18). These T cells may also participate in auto-immune responses against bone antigens or in the activation of hematopoietic stem cells. Moreover, the cytokine production profile of OCLs is not only dependent on their physiological or inflammatory environment and cell origin (18) but also on their capacity to respond to bone and bone matrix proteins by secreting high levels of the pro-inflammatory cytokine IL-1β (83, 85, 86, 209). Overall, these data point inflammatory OCLs as major actors in a vicious circle linking bone destruction and inflammation.

This contribution of OCLs not only to bone resorption and homeostasis, but also to immune responses, encompasses the function of classical OCLs and sheds new light on the field of osteoimmunology. These insights make OCL an important target for anti-inflammatory therapies of chronic inflammatory diseases as well as for influencing the bone environment.

Of note, pathologies related to abnormal OCL activity/differentiation are frequently associated with immune dysfunctions not only in mouse but also in human. In osteopetrotic patients with defective OCL activity or differentiation, bone marrow failure is responsible for extramedullary hematopoiesis and contributes to immune deficiency and increases the risk of infections (210, 211). Besides, genes affected by osteopetrotic defects are not only essential for bone resorption but are also involved in immune responses. Among other examples, deficiency in *Acp5* (encoding TRAcP) induces bone dysplasia but also autoimmunity (212, 213) and deletion or inhibition of *Ctsk* blocks both bone degradation and inflammation (214, 215). In osteoporosis, a number of immune-system related genes are differentially expressed in the BM of post-menopausal osteoporotic patients compared to non-osteoporotic individuals, including cytokines and factors involved in innate immunity (216–218). While such differences in gene expression and immune cell activation are clearly promoting bone destruction, the participation on OCLs in these immune modifications is still not known but cannot be excluded.

The immune function of OCLs emerged only recently, explaining why its contribution to OCL-associated diseases or its modulation by anti-resorption therapies has not yet been explored. A major issue for such investigation is the absence of markers specific for human OCL subsets that are required to identify inflammatory and anti-inflammatory OCLs. Thus, a deeper molecular profiling of human OCL sub-populations and characterization of their immune function remains essential to enable further studies in affected patients.

## Conclusion

In the last decade, remarkable advances have been made in understanding the interactions between the skeletal and the immune system under both physiological and pathological conditions. In particular, the influence of T cells on OCL formation and activation through complex cytokine interactions including TNFα and RANKL were thoroughly investigated and immune cells were shown to regulate bone cell differentiation and activity. Today however, these interactions are known to be reciprocal, increasing further, the interest for OCLs as immune cells.

Depending on the context, different OCLs are described to derive from distinct progenitor cells. Based on the numerous OCL precursors described, and on the recent identification of an iterative fusion of mature OCLs with circulating monocytic cells^2^ (38), the possibility of OCL heterogeneity is huge. Additionally, some precursor cells seem to differentiate much more easily than others depending on their context. The existence of heterogeneous OCL populations appears unsurprising when considering that OCL precursors, including MNs and DCs, have been described as phenotypically and functionally heterogeneous for many years. Thus, bone destruction does not rely only on an increase in OCL differentiation and function, but also on the recruitment of OCL subsets that differ from steady state OCLs.

These novel insights in the field of osteoimmunology open new exciting perspectives and emphasize that OCL function is not restricted to bone resorption but expanded to immune cell differentiation and immunomodulation. Based on these observations and according to their immune function, OCLs could act as key players and regulators of the bone immune status in steady state as well as during inflammatory processes and they should not anymore be regarded only as bone-resorbing cells. Therefore, relying only on bone resorption may not be sufficient to block inflammatory bone destruction. New specific anti-resorptive agents targeting inflammatory OCLs and the associated T cell interaction could provide a very novel effective strategy to control inflammatory bone loss and the bone environment without compromising physiological bone remodeling by steady state OCLs.

## Author Contributions

CB-W conceived the original idea and designed the project. M-BM, LI, TdV, AW, FA, and AT participated in writing the manuscript. M-BM and LI designed the figures. M-BM edited the figures. CB-W edited the manuscript.

### Conflict of Interest Statement

The authors declare that the research was conducted in the absence of any commercial or financial relationships that could be construed as a potential conflict of interest.

## Footnotes

^1^https://www.armchairmedical.tv/anz-bone-mineral-society/videos/intravital-imaging-of-osteoclasts-in-vivo-reveals-a-novel-cell-fate-mechanism-dr-michelle-mcdonald

^2^https://www.armchairmedical.tv/anz-bone-mineral-society/videos/intravital-imaging-of-osteoclasts-in-vivo-reveals-a-novel-cell-fate-mechanism-dr-michelle-mcdonald
